# Antifungal Susceptibility of *Saccharomyces cerevisiae* Isolated from Clinical Specimens

**DOI:** 10.3390/pathogens13030248

**Published:** 2024-03-14

**Authors:** Aleksandra Górzyńska, Kamila Kondracka, Agnieszka Korzeniowska-Kowal, Urszula Nawrot

**Affiliations:** 1 Department of Pharmaceutical Microbiology and Parasitology, Wroclaw Medical University, 50-556 Wroclaw, Poland; kamila_kondracka@onet.pl (K.K.); urszula.nawrot@umw.edu.pl (U.N.); 2 PhD School of Wroclaw Medical University, Wroclaw Medical University, 50-345 Wroclaw, Poland; 3 Department of Immunology of Infectious Diseases, Hirszfeld Institute of Immunology and Experimental Therapy, Polish Academy of Sciences, St. Weigla 12, 53-114 Wroclaw, Poland; agnieszka.korzeniowska-kowal@hirszfeld.pl

**Keywords:** *Saccharomyces cerevisiae*, azoles, echinocandins, amphotericin B, flucytosine, manogepix

## Abstract

(1) Background: Despite being considered a non-pathogenic yeast, recently, a growing occurrence of *Saccharomyces cerevisiae* infections has been noted. There is little knowledge about the drug susceptibility of this species. Therefore, the objective of this research was to expand it and determine the drug susceptibility profile of a local collection of clinical isolates of this species. (2) Methods: This study contained 55 clinical isolates identified as *Saccharomyces cerevisiae* using the MALDI-TOF method. The susceptibility of *Saccharomyces cerevisiae* was tested to 10 antifungals (amphotericin B, flucytosine, fluconazole, voriconazole, posaconazole, micafungin, anidulafungin, caspofungin, and itraconazole) using MICRONAUT-AT tests and manogepix, a new drug, using the microdilution method according to EUCAST. (3) Results: Overall, most strains were classified as sensitive to amphotericin B and flucytosine (MIC ranges of ≤0.03–1 and ≤0.06–0.125, respectively) and also to echinocandins. However, five isolates expressed high MIC values for all of the tested azoles, indicating cross-resistance. The MIC range for manogepix was 0.001–0.125 mg/L, with an MIC_50_ of 0.03 mg/L and an MIC_90_ of 0.06 mg/L. (4) Conclusions: The occurrence of resistance to azoles may be a concerning problem and therefore should be investigated further. However, the new antifungal manogepix appears to be an interesting new therapeutic option for treating such infections.

## 1. Introduction

*Saccharomyces cerevisiae* is a yeast species deeply rooted in the age-old practices of bread baking and fermented beverage production [1]; it assumes a dual role as an indispensable contributor to the fermentation industry and a cornerstone in scientific research, serving as an experimental model organism [2]. Its significance transcends into culinary applications, as it has emerged as a versatile model for probing various biological processes, ranging from metabolism, aging, apoptosis, and gene expression regulation to the intricacies of the cell cycle, signal transduction, and investigations into neurodegenerative disorders [3].

Beyond its scientific value, *Saccharomyces cerevisiae* has also found use as a dietary supplement. Recognized for having many essential nutrients, including zinc, selenium, phosphorus, magnesium, chromium, B vitamins, amino acids, and proteins, it boasts a comprehensive nutritional profile. These dietary supplements are often recommended for individuals coping with weakness, fatigue, or issues related to skin, hair, and nails. Moreover, they serve as a supportive measure for people in the recovery phase after an illness or surgery [4,5,6].

Despite its non-pathogenic association, there is a growing prevalence of *S. cerevisiae* isolation from various human anatomical sites, including the gastrointestinal, respiratory, and genital tracts, driven by emerging reports of infections caused by this once-considered benign species [4,7]. While the frequency of the most severe infections, such as fungemia caused by *S. cerevisiae* yeast, remains unknown, it is estimated that they may account for 0.1–3.6% of all cases of bloodstream fungal infections [7].

Infections associated with *S. cerevisiae* span from organ-specific to generalized mycoses, including fungemia, endocarditis, liver abscesses, and pneumonia. Vulnerable populations involve mostly premature infants, individuals over 60, and those under immunosuppression or intensive care, and instances of infection have also been documented in immunocompetent patients [7]. The infection routes involve translocation from the intestine to the bloodstream, dissemination to other organs, and catheter-related infections, particularly central venipuncture [7]. A notable risk factor is the intake of probiotics containing *S. cerevisiae* var. *boulardii*, surpassing the cumulative incidence of infections caused by probiotic bacteria [8]. Therefore, meticulous analysis of yeast probiotic usage is warranted, especially in intensive care units among catheterized patients receiving enteral nutrition and those undergoing broad-spectrum antibiotic therapy [7]. A comprehensive analysis of a patient’s condition, considering age and comorbidities, is essential before recommending probiotics containing *Saccharomyces cerevisiae* var. *boulardii* [7].

Despite the pathogenicity associated with yeast probiotics, *S. cerevisiae* var. *boulardii* exhibits advantages as a probiotic. Its resistance to gastric conditions allows it to reach the intestine untouched, showing efficacy in post-antibiotic diarrhea, travelers’ diarrhea, and diarrhea associated with enteral nutrition. Additionally, it proves beneficial in managing infections caused by *Helicobacter pylori* and *Clostridium difficile*, presenting a potential role in reducing exacerbations of inflammatory bowel disease, including ulcerative colitis and Crohn’s disease. Moreover, this probiotic demonstrates effectiveness in alleviating symptoms and improving the well-being of patients with irritable bowel syndrome (IBS) [9,10].

The fight against *S. cerevisiae* infections is intricate and contingent significantly on the susceptibility profile of the strain. Commonly employed antifungal agents, such as fluconazole and amphotericin B, have played a main role in treatment. However, studies on the susceptibility profile of *S. cerevisiae* to antifungal drugs remain scarce, with noteworthy high minimum inhibitory concentration (MIC) values for fluconazole and itraconazole [8]. Caspofungin’s good in vitro activity is suggested to be an alternative for treating *S. cerevisiae* infections [8].

The primary objective of this study was to investigate the susceptibility of *Saccharomyces cerevisiae* to antifungal drugs, including the novel antifungal agent manogepix. The reference microdilution method, considered the most reliable approach for determining fungal drug susceptibility, served as the foundation for this investigation. This method is also a base for constructing commercial tests, with readings based on either turbidity (visually or spectrophotometrically by measuring extinction) or metabolic activity (visually or spectrophotometrically). 

In the present study, the susceptibility of *Saccharomyces cerevisiae* to nine antimycotics—amphotericin B (AmB), flucytosine (FC), fluconazole (FLU), voriconazole (VOR), posaconazole (POS), itraconazole (ITR), anidulafungin (AND), micafungin (MFI), and caspofungin (CAS)—was assessed using MICORNAUT-AT assays, and manogepix’s effectivity was examined by using the microdilution method according to EUCAST [11].

The mechanism of action of manogepix (MGX) functions by inhibiting the fungal Gwt1 enzyme [12,13]. This inhibition results in pleiotropic effects on the fungal cell, such as impairment of cell wall mannoprotein localization, compromised cell wall integrity, hindered biofilm formation, and impaired germ tube formation, causing inhibition of fungal growth [13,14].

Notably, MGX exhibits broad in vitro activity against major fungal pathogens, such as the *Candida* (including *Candida auris*), *Cryptococcus*, *Aspergillus*, *Scedosporium*, and *Fusarium* species [14,15]. Moreover, its efficacy extends to include azole-resistant and echinocandin-resistant strains of *Candida* and *Aspergillus*, demonstrating sustained activity both in vitro and in vivo [14,16]. This versatility positions MGX as a promising antifungal agent with potential applications in combating a diverse range of fungal infections, particularly those characterized by resistance to conventional antifungal treatments. 

This article aims to enhance our understanding of *S. cerevisiae* strains isolated from humans, encompassing their susceptibility to antimycotics and growth characteristics on selected microbiological media. By delving into the intricate world of antifungal susceptibility testing, we aim to enlighten current susceptibility to commonly used antifungal drugs. This exploration not only advances comprehension of the clinical relevance of *S. cerevisiae*, but also establishes a foundation for more targeted and efficacious treatment modalities within the dynamic field of medical mycology.

## 2. Materials and Methods

### 2.1. Microorganisms and Culture

This study included 55 *Saccharomyces cerevisiae* strains: 51 clinical isolates from University Clinical Hospital in Wroclaw (feces and rectal swabs: 31, upper respiratory tract swabs: 6, lower respiratory tract materials: 7, blood: 6, genital tract swabs: 3, urine: 2, pus: 1, ear swab: 1), 2 isolates from food products (baker’s yeast and kefir), and 1 obtained from the commercial dietary supplement Enterosive (Silesian Pharma, Katowice, Poland), which, according to the manufacturer, contains *Saccharomyces boulardii* CBS 5926. The strains *Saccharomyces cerevisiae* BCCM/IHEM 3963 (Belgian Coordinated Collection of Microorganisms/Fungi Collection: Human & Animal Health) and *Candida krusei* ATCC 6258 (American Type Culture Collection) were used as references. *Saccharomyces* strains were stored at −75 °C in TSB liquid medium with glycerol. After defrosting, the strains were inoculated onto Sabouraud Dextrose LAB-AGAR™ (SGA) (BioMaxima, Lublin, Poland) agar medium and incubated at 37 °C and 28 °C for 24–48 h. The purity of the grown strains was evaluated by screening on Chromagar Candida (Becton Dickinson, Franklin Lakes, NJ, USA) medium, and they were subjected to MALDI-TOF (Matrix-Assisted Laser Desorption Ionization Time-of-Flight) for further identification.

In addition, a test of the ability of strains to multiply in the media recommended for susceptibility testing (a liquid medium RPMI-1640 (Sigma-Aldrich, St. Louis, US-MO, USA) supplemented with 2% glucose and buffered with MOPS (3-(N-Morpholino)propanesulfonic acid), Sigma-Aldrich, St. Louis, US-MO, USA) as well as a Mueller–Hinton agar supplemented with glucose and methylene blue (MH + GMB) (BioMaxima, Lublin, Poland) was performed. The 24 h yeast cultures on SGA agar were suspended in sterile distilled water to a density of 0.5 on the McFarland scale, which was then diluted 10 times. Then, 50 µL of each inoculum was transferred to a 96-well microtiter plate. Then, 50 µL of twice-concentrated RPMI-1640 medium (2× RPMI-1640) was added and the whole plate was incubated at 37 °C for 24–48 h. At the same time, the same strain suspension was inoculated with a sterile swab onto MH + GMB agar medium and also incubated, under the same conditions.

### 2.2. MALDI-TOF Performance

To perform MALDI-TOF identification, ribosomal proteins were isolated from each strain via extraction with ethanol and formic acid to obtain high-quality mass spectra. For extraction, one to five colonies were collected from fresh cultures incubated for 24 h on Saboraud agar at 37 °C. The collected material was suspended in 300 µL of distilled water. Then, 900 µL of pure ethanol was added and the samples were homogenized using a vortex-type device for 30 s and centrifuged for 2 min at 13,000× *g*. After removing the supernatant, the precipitate was dried at room temperature. Next, 25 µL of 70% formic acid solution was added and the sample was thoroughly mixed by pipetting. Subsequently, 25 µL of acetonitrile was added and mixed again. The samples underwent centrifugation for 2 min at 13,000× *g*. For further analysis, 1 µL of the supernatant was applied to a steel plate (MSP 96-target polished steel BC plate) and allowed to dry for 15 min at room temperature. Then, 1 µL of HCCA (α-Cyano-4-hydroxycinnamic acid) matrix was applied to the spotted, dried supernatant and allowed to dry once again. This way, the prepared plate was loaded into a MALDI Biotyper Sirius mass spectrometer (Bruker, Billerica, MA, USA laser frequency: 200 Hz) and mass spectra of the extracted ribosomal proteins were obtained using flexControl Version 3.4 software. Each mass spectrum was generated by averaging 1500 laser shots from three positions. The spectra underwent calibration using the *E. coli* DH5-alpha standard (Bruker). MBT Compass 4.1 and MALDI Biotyper Compass Explorer 4.1 software were used to identify the mass spectra, as well as the MBT Compass Library Revision H (2021) database (Bruker Daltonics GmbH & Co. KG, Bremen, Germany), containing 3893 species. 

As per the manufacturer’s instructions, the identification results are classified as follows:

2.300–3.000: Highly probable species identification.

2.000–2.299: Safe genus identification, probable species identification. 

1.700–1.999: Probable genus identification.

0.000–1.699: No identification.

### 2.3. MICRONAUT-AT Testing

Drug susceptibility testing was conducted using MICRONAUT-AT (MERLIN Diagnostic GmbH, Berlin, Germany) tests, which included pre-prepared 96-well microtiter plates containing nine lyophilized antimycotics in a concentration gradient (amphotericin B, flucytosine, fluconazole, voriconazole, posaconazole, micafungin, anidulafungin, caspofungin, and itraconazole). Each test set also featured tubes of 11.5 mL colorless RPMI-1640 liquid medium, along with two indicators: an AST-indicator and methylene blue.

Yeast suspensions were standardized to a density of 0.5 McFarland in 4 mL of NaCl. Following this, 10 µL of the prepared fungal suspension was added to 11.5 mL of RPMI-1640. To this mixture, 100 µL of the AST indicator (resazurin) and 50 µL of methylene blue solution were introduced. Using a multichannel pipette, 100 µL of the suspension was added into each of the 96 wells on the plate. The plate was sealed and then incubated at 35 °C for 24 h.

Results were visually examined and interpreted according to the manufacturer’s guidelines. A blue color indicated no growth of the strain, while pink denoted growth, and a colorless appearance signified enhanced growth. 

### 2.4. Microdilution Reference Method According to EUCAST

To test susceptibility to manogepix, the microdilution method following EUCAST guidelines (The European Committee on Antimicrobial Susceptibility Testing) [11] was employed. 

For this purpose, 96-well sterile microplates containing a gradient of antimycotic concentrations were meticulously prepared. In this context, 50 µL of the drug’s working liquid solution in 2× RPMI-1640 medium was applied. Each plate comprised eight dilution series of the examined substance, tailored to test the susceptibility of eight strains. The prepared titration plates were methodically stored at −75 °C until utilization.

Cultures of the test strains, grown for 24 h, were resuspended in sterile distilled water to achieve a McFarland density of 0.5. Subsequently, the suspensions underwent a 10-fold dilution in sterile distilled water. After removing the plates from freezing storage and bringing them to room temperature, 50 µL of the prepared suspensions of the test strains was applied to all 96 wells, attaining the recommended inoculum density of 0.5–2.5 × 10^5^ CFU/mL. The plate was incubated for 24 h at 35 °C, and absorbance results were read using a Multiskan Go (ThermoFisherScientific, Waltham, MA, USA) microplate reader at 530 nm.

The MIC result reading was established as the lowest concentration of MGX at which the absorbance result equaled or was less than 50% of the absorbance obtained for the growth control of the isolate. 

## 3. Results

The analysis performed with the use of the MALDI-TOF method confirmed the identification of all investigated *Saccharomyces cerevisiae* isolates, with MALDI scores ranging between 1.86 and 2.16. Comprehensive data for each isolate are included in Table S1 (Supplementary Materials). Six clinical isolates of *Saccharomyces cerevisiae* did not grow on the RPMI-1640 medium and for this reason the susceptibility test included only 55 isolates, of which incubation had to be extended to 48 h for 3. The ability to grow in the synthetic media was also included in Table S1. 

**Figure 1:**
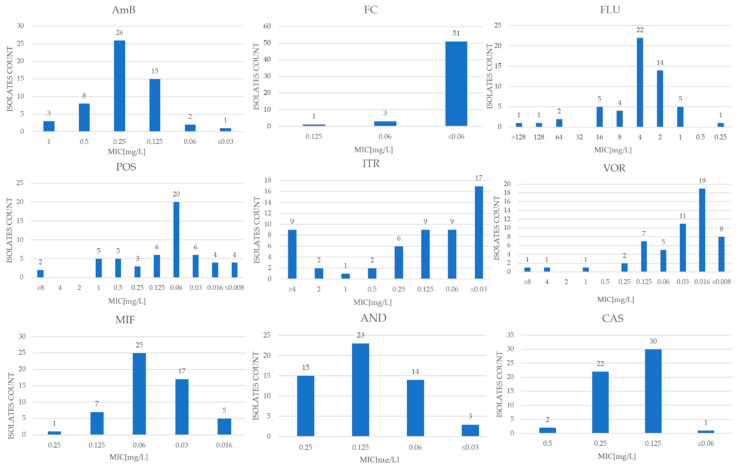
The distribution of MIC values of nine antimycotics for *Saccharomyces cerevisiae* strains obtained through the use of MICRONAUT-AT tests.

Due to the lack of designated clinical cutoff values (CBP) for *S. cerevisiae* species, preliminary criteria described in the scientific literature and CBP values set by EUCAST for *Candida albicans* and *Candida glabrata* were used to interpret the results, except for amphotericin B and itraconazole. EUCAST established ECOFF as 0.5 and 2 mg/L, respectively, for these antifungals [17]. The MIC (minimum inhibitory concentration) values and MIC_50_ and MIC_90_ for each antifungal are shown in Table 1 and also in Figure 1. To ensure that the test was authoritative, it was performed using the reference strains *Saccharomyces cerevisiae* BCCM/IHEM 3963 and *Candida krusei* ATCC 6258. Regarding the EUCAST quality-control isolate *Candida krusei* ATCC 6258, all observed results were consistent with the MIC range established by EUCAST for this reference strain. The results for these quality-control strains were closely presented in Table 1.

In the MICRONAUT-AT tests, the MIC values of amphotericin B for the tested strains oscillated between <0.003 and 1 mg/L. In contrast, the growth-inhibitory concentration of 50% of the tested microbial population (MIC_50_) for AmB was 0.25 mg/L, and the growth-inhibitory concentration of 90% of the strain population (MIC_90_) was 0.5 mg/L. The epidemiological cutoff value (ECOFF) published by EUCAST is 0.5 mg/L [17]. Based on this, 52 examined strains were designated as WT (wild-type), while 3 were classified as N-WT (non-wild-type).

The MIC results for flucytosine were <0.06–0.125 mg/L, while MIC_50_ was <0.06 mg/L and MIC_90_ was <0.06 mg/L. Assuming a value of ≤1 mg/L for sensitive strains [18], all clinical isolates (100% of the study population) were classified as sensitive.

The lowest inhibitory concentration for anidulafungin was between 0.03 and 0.25 mg/L, with an MIC_50_ value of 0.125 mg/L and an MIC_90_ of 0.25 mg/L. Assuming a cutoff value of ≤0.5 mg/L [18] for the determination of sensitive strains, the entire study population of 55 clinical isolates was classified as S (sensitive).

For caspofungin and micafungin, the MIC values took values of 0.06–0.5 mg/L and 0.16–0.25 mg/L, respectively, with MIC_50_ and MIC_90_ values of 0.125 mg/L and 0.06 mg/L, and 0.25 mg/L and 0.125 mg/L, respectively.

The MIC values of fluconazole for the clinical isolates ranged from 0.25 to >128 mg/L, while MIC_50_ was 4 mg/L and MIC_90_ was 16 mg/L. Assuming an MIC of ≤4 mg/L for sensitive strains [19], 42 clinical isolates (76% of the study population) were classified as sensitive (S), while 13 test strains (around 24%) were classified as potentially resistant (R). 

For voriconazole, MIC values were between <0.008 and >8 mg/L, with an inhibitory concentration for 50% of the tested population of strains of 0.016 mg/L, and an inhibitory concentration for 90% of the population of 0.125 mg/L. An MIC value of ≤0.5 mg/L [18] or ≤0.25 mg/L [20,21] was used as the initial cutoff point for susceptible strains. For both criteria, 52 samples (94.5% of the study population) were classified as susceptible. The lowest inhibitory concentrations for itraconazole ranged from <0.03 to >4 mg/L, with an MIC_50_ value of 0.125 and MIC_90_ of >4 mg/L. Using the ECOFF value of 2 mg/L published by EUCAST [22], 43 strains (78% of the population) fell into the WT group, and 12 fell into the N-WT population. For posaconazole, the MIC range was <0.008 to >8 mg/L, with an MIC_50_ of 0.06 mg/L and an MIC_90_ of 1 mg/L.

**Figure 2:**
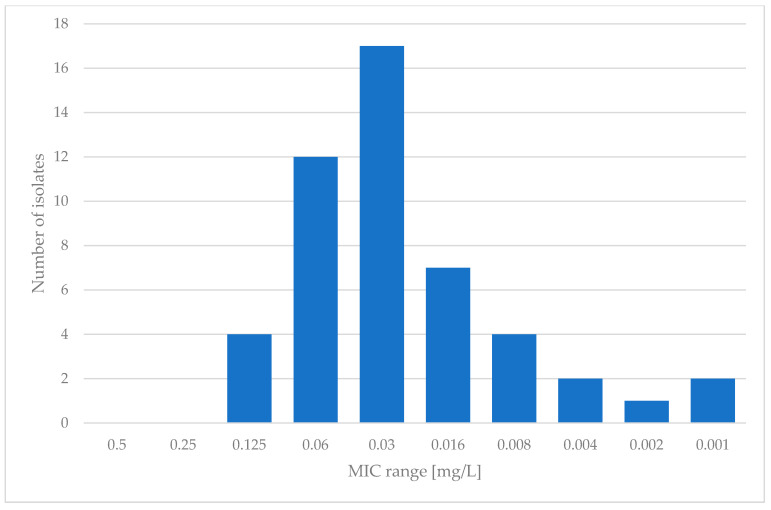
Susceptibility of clinical isolates of *S. cerevisiae* to manogepix.

Regarding MGX, the microdilution method according to EUCAST was employed. Examination was limited to 49 isolates that showed sufficient growth in RPMI-1640 medium after 24 h of incubation. For manogepix, the MIC range varied from 0.001 to 0.125 mg/L, with an MIC_50_ of 0.03 mg/L and an MIC_90_ of 0.06 mg/L. The results for manogepix are presented in Figure 2.

## 4. Discussion

The documented increase in fungal infections, particularly in developed countries, has drawn attention to infections caused by *Saccharomyces cerevisiae*, formerly considered as non-pathogenic [7]. There is a growing number of reports on invasive infections (mostly fungemia) caused by *Saccharomyces cerevisiae*, including *S. boulardii*, a variant that can be found in probiotic preparations. Limited data in the literature exist regarding the drug susceptibility profile of these microorganisms, which can potentially cause challenges in the interpretation of the results [19]. The primary objective of this study was to investigate the susceptibility of *S. cerevisiae* to antifungal drugs. MICORNAUT-AT commercial tests, which are customized to both CLSI and EUCAST standards, were employed to assess susceptibility to nine antimycotics (AmB, FC, CAS, AND, MFI, FLU, ITR, VOR, POS). A limitation encountered in this study was that the population of strains tested was not screened for the *S. boulardii* variant. Unfortunately, due to the high genetic similarity between *Saccharomyces cerevisiae* and its “*boulardi*” variant, it is impossible to distinguish them using gold-standard methods based on ribosomal DNA sequences and more comprehensive genetic analysis is required (e.g., determining the absence of genes for the hexose transporters HXT11 and HXT9 and the use of asparagine), as was performed in a study from 2017 by Indu Khatri et al. [20]. Therefore, the MALDI-TOF method used in this study unfortunately did not differentiate precisely between *Saccharomyces cerevisiae* and *Saccharomyces cerevisiae* var. *boulardi*. This distinction would be particularly important in relation to strains isolated from blood samples, as the pathogenic properties of *S. boulardii* still seem insufficiently documented. We plant to implement this task in future studies. 

However, another unforeseen challenge emerged during this research, particularly with the growth of some *S. cerevisiae* strains on synthetic media. Strains identified as *S. cerevisiae* species underwent growth control on RPMI-1640 and MH + GMB media, revealing that six strains lacked the ability to grow on MH + GMB medium. Regarding the RPMI-1640 medium, 15% of the tested pool of strains showed delayed growth (72 h) or no growth. These strains were most likely auxotrophs, requiring additional factors not provided by the aforementioned media. A similar effect was observed by Barchiesi et al. [21], wherein 20% of isolates failed to grow on RPMI-1640 medium. What is interesting is that most of the strains that exhibited delayed growth on synthetic media in this study and also in the study by Barchiesi [21] were isolated from the gastrointestinal tract. This may indicate that they were non-pathogenic and could be part of the temporary or permanent intestinal microbiota. What raises attention, however, is the fact that the probiotic isolates from ready-to-use formulations (such as kefir or Enterosive, a dietary supplement) used in this study all expressed the ability to grow in both media. However, according to our knowledge, neither EUCAST nor CLSI report growth difficulty with this yeast. The lack of growth of clinical isolates may pose a significant challenge in establishing their susceptibility to antifungal drugs. Taking all factors into consideration, finally, 55 isolates were included in this study and the MICRONAUT-AT test was conducted.

An additional difficulty encountered during the interpretation of the results was the fact that for the *S. cerevisiae* species, there are no clinical breakpoints established by scientific societies for assessing sensitivity, except for AmB and ITR, for which EUCAST has set epidemiological cutoff values (ECOFFs) of 0.5 mg/L and 2 mg/L, respectively [22]. Most authors adopt AmB limits for *Candida* at 1 mg/L. According to this criterion, 100% of the test strain population exhibited sensitivity to amphotericin B (MIC range ≤ 0.03–1 mg/L), aligning with a similar study [18], where only 0.4% of *S. cerevisiae* isolates were found to be resistant to AmB (MIC range ≤0.03–2 mg/L) (Table 2).

In the case of FC, no interpretative criteria for any yeast species are included in current EUCAST recommendations. Previous research has suggested a value of ≤0.5 mg/L for susceptible strains, which was the criterion applied in this study, leading to 100% of strains (MIC range ≤0.06–0.125 mg/L) being described as sensitive. This differs from a study conducted by Borman et al. [18], wherein 16.7% of the tested population was classified as potentially resistant (MIC range ≤0.125–>64 mg/L). Perhaps this difference may be caused by the fact that the tested pull was significantly larger and contained 626 isolates of *Saccharomyces cerevisiae*. However, another issue is that resistance to FC evolves rather easily, which was presented in the research by Durand and al. [23]. In their study, several resistant S. *cerevisiae* mutants were tested, and what is interesting is that these authors showed that resistance was either acquired through pleiotropic drug response, marked by cross-resistance to fluconazole, or by loss-of-function mutations in FUR1, which encodes an important enzyme in the metabolism of 5-FC. Some of the tested strains came from the environment which, as authors state, may suggest that ecological interactions may dictate the identity of resistance hotspots.

The next group of tested antifungals were echinocandins, which currently are a primary choice for treating candidemia and invasive candidosis due to their high efficiency against *Candida* spp., including azole-resistant strains, weak drug interactions, and a low toxicity profile compared to azoles [24]. In this research, three echinocandins were studied: micafungin, anidulafungin, and caspofungin. The biggest issue may concern caspofungin, since EUCAST does not recommend using the microdilution method in this particular case. Due to low repeatability of the results and significant interlaboratory variation in MIC ranges for this drug, EUCAST does not state the limit values and advises the use of breaking points for micafungin and anidulafungin for interpretation of the results [25]. In this study, caspofungin was included and the results were interpreted through susceptibility to MIF and AND. Concerning caspofungin, the MIC values ranged from 0.06–0.5 mg/L, lower than the range reported by Desnos-Ollivier et al. [26] (0.5–1 mg/L). These authors established both MIC_50_ and MIC_90_ to be 1 mg/L, significantly higher than the values found in this study, which were 0.125 and 0.25 mg/L, respectively. Similarly, for micafungin, the MIC range obtained in this study was slightly lower than the values reported by Desnos-Ollivier et al. [26] (Table 1 and Table 2).

However, the clinical interpretation of clinical breakpoint (CBP) values for anidulafungin exhibits significant variation across fungal species. For instance, *C. parapsilosis*, with naturally high MIC values, has an accepted CBP for anidulafungin at 4 mg/L, while for *C. albicans*, it is 0.03 mg/L [22]. For the tested *S. cerevisiae* strains, a proposed MIC value of ≤0.5 mg/L was suggested [18,27]. Applying this criterion would classify all strains as sensitive, aligning with results reported by Borman et al. [18], where only 0.7% of the tested isolates were found to be N-WT. 

The MIC values obtained for AmB, FC, and echinocandins in the MICRONAUT testing align with other studies on strains of this species; however, MIC values of azole drugs using the MICRONAUT-AT test are more problematic due to the trailering phenomenon, which hinders complete inhibition of fungal growth at increasing concentrations of antimycotics [28]. 

During this research, a difficulty was observed, most likely due to errors in the establishment of the inoculum, where the reading after 24 h was followed by discoloration, indicating resistance, but in subsequent repetitions performed by another person from the research team, this possibility was ruled out. This may present a difficulty in performing this test on a daily basis. Attention should be paid to this and great care should be taken in preparing the test and also in interpretating the results, since interpretations are based on color change. Even though the test was prepared according to the manufacturer’s instructions, it is essential to carefully prepare the inoculum, because the amount recommended in the instruction is very little (10 µL) and can be hard to apply, which may cause difficulty for the person performing the test. In this study, four azoles were tested: fluconazole, itraconazole, voriconazole, and posaconazole.

Regarding fluconazole, the final MIC range was 0.25–≥128 mg/L, with 92.7% of strains being considered as susceptible at an MIC of ≤16mg/L. This contradicts the results reported by Borman et al. [18], where only 56.9% of *Saccharomyces cerevisiae* isolates were established as sensitive to fluconazole. However, these authors tested over 600 isolates and MIC_90_ was 16 mg/L, the same as the one presented in this study.

Considering itraconazole (ITR), the MIC cutoff value was between ≤0.03 and ≥4 mg/L, with MIC_50_ and MIC_90_ at 0.125 and ≥4 mg/L, respectively. The recently published ECOFF by EUCAST (2 mg/L) classified 16.4% of isolates as non-wild-type (N-WT). In the study by Borman et al. [18], the percentage of resistant isolates was much higher and was stated as 37%. 

For voriconazole and posaconazole, MIC values ranged between ≤0.008 and ≥8, with MIC_50_ and MIC_90_ being 0.016 and 0.06 mg/L and 0.125 and 1 mg/L, respectively. Resistance to voriconazole was established at 5.5%, slightly surpassing the results from Borman et al. [18], where resistance was stated for 4% of tested isolates. In the case of posaconazole, the MIC values reached ≥8 mg/L, exceeding the values reported by Desnos-Ollivier et al. [26], highlighting the need for further investigation into susceptibility patterns. What is interesting is the fact that in the case of the azoles, a cross-resistance can occur. Two isolates (No. 523 and No. 824: Table S1, included in the Supplementary Materials) expressed really high MIC values for all of the azoles tested in this study. Overall, the MIC values of five isolates indicated azole resistance, while two of the tested strains turned out to be only itraconazole-resistant, with susceptibility for at least other two azoles. However, the growth inhibition initiated by each antimycotic was visually determined after 24–48 h of incubation at 37 °C, with reagents resazurin, metabolized by living cells, and methylene blue, helpful in visually assessing growth inhibition based on color change. The difficulty with establishing the actual color that indicated the lack of growth probably depended on the amount of dye reactant applied. Therefore, a careful preparation of the test according to the manufacturer’s instructions must be maintained. 

When it comes to manogepix, not much research has been conducted. It is quite hard to find data concerning the susceptibility of *Saccharomyces cerevisiae* in the literature. However, in extensive research conducted by Faller et al. [29] in 2021, which contained many different yeast species, nine isolates of *Saccharomyces cerevisiae* were tested. These authors stated MIC values between 0.008 and 0.06 mg/L, similar to those in the present study. The MIC values presented in this study ranged between 0.001 and 0.125 mg/L, which matches the results gained by the authors mentioned above. During our research, we encountered a difficulty with growth and reading of the results for some isolates. Therefore, the presented data concern only the isolates whose growth allowed the results to be read unproblematically. However, the susceptibility to manogepix should be investigated further due to the lack of data. In Table 2, all data are presented and compared with the results of the other studies mentioned in this paper.

**Table 2:** Susceptibility profiles of *Saccharomyces cerevisiae* data published by various authors.

Antifungal	[ref]	n	MIC Range [mg/L]	MIC_50_ [mg/L]	MIC_90_ [mg/L]	CBP	S (%)	R (%)
AmB	[18]	448	≤0.03–2			1		0.4
	[27]	58	0.03–1					
	[p. r.]	55	≤0.03–1	0.25	0.5	1	100	0
						0.5	94.5	5.5
FC	[18]	168	≤0.125–>64			1		16.7
	[p. r.]	55	≤0.06–0.25	≤0.06	≤0.06	1	100	0
CAS	[30]	21	0.5–1	1	1			
	[p. r.]	55	0.125–0.5	0.125	0.25			
AND	[18]	97	≤0.015–2			0.5		0.7
	[27]	63	0.016–0.5			0.5		
	[p. r.]	55	≤0.03–0.25	0.125	0.25	0.5	100	0
MIF	[30]	21	0.12–0.5	0.25	0.25			
	[p. r.]	55	0.016–0.25	≤0.06	0.125			
ITR	[18]	416	≤0.03–32			0.5		37
	[p. r.]	55	≤0.03–≥4	0.125	≥4	2	83.6	16.4
VOR	[18]	323	≤0.03–4			0.5		4
	[27]	48	0.06–1			0.25		
	[26]	61	≤0.015–1	0.125	0.25			
	[p. r.]	55	≤0.008–≥8	0.016	0.125	0.5	94.5	5.5
						0.25	94.5	5.5
POS	[26]	61	≤0.015–4	0.5	1			
	[p. r.]	55	≤0.008–≥8	0.06	1			
FLU	[18]	612	≤0.125–>64			4		43.1
	[27]	64	2–≥32			16		
	[26]	61	≤0.125–32	8	16			
	[30]	21	2–8	4	8			
	[p. r.]	55	0.25–≥128	4	16	4	76	24
						16	92.7	7.3
MGX	[29]	9	0.008–0.06					
	[p. r.]	49	0.001–0.125	0.03	0.06			

Abbreviations: [p. r.]—“personal research”—data published in this study.

## 5. Conclusions

*Saccharomyces cerevisiae* can be relatively often isolated from clinical materials, although they rarely cause an infection. The presented study allowed us to expand the existing knowledge regarding the susceptibility of *Saccharomyces cerevisiae* to commonly used antifungal drugs and indicates that clinical isolates are mostly susceptible. The occurrence of resistance to azoles may be a concerning problem and therefore should be investigated further. However, the new antifungal manogepix appears to be a promising treatment option due to the high susceptibility of *Saccharomyces cerevisiae.*

## Figures and Tables

**Table 1:** A summary of MIC ranges and MIC_50_ and MIC_90_ values for the investigated *S. cerevisiae* strains compared to reference strains *Saccharomyces cerevisiae* BCCM/IHEM 3963 and *Candida krusei* ATCC 6258.

Antifungal	MIC [mg/L] Range	MIC_50_	MIC_90_	*Saccharomyces cerevisiae* BCCM/IHEM 3963	*Candida krusei* ATCC 6258
AmB	≤0.03–1	0.25	0.5	0.06	0.25
FC	≤0.06–0.125	≤0.06	≤0.06	≤0.06	4
CAS	0.06–0.5	0.125	0.25	0.06	0.125
AND	0.03–0.25	0.125	0.25	0.06	0.03
MIF	0.016–0.25	≤0.06	0.125	0.03	0.03
FLU	0.25–≥128	4	16	2	32
ITR	≤0.03–≥4	0.125	≥4	0.25	0.03
VOR	≤0.008–≥8	0.016	0.125	0.016	0.06
POS	≤0.008–≥8	0.06	1	0.06	0.03

Abbreviations: *Saccharomyces cerevisiae* BCCM/IHEM 3963—control strain *Saccharomyces cerevisiae* BCCM/IHEM 3963 (Belgian Coordinated Collection of Microorganisms/Fungi Collection: Human & Animal Health); ATCC 6258—control strain *Candida krusei* ATCC 6258 (American Type Culture Collection).

## Data Availability

The original data are presented in the article and in the Supplementary Materials (Table S1). For more information, please contact the corresponding author.

## Supplementary Materials

The following supporting information can be downloaded at https://www.mdpi.com/article/10.3390/pathogens13030248/s1: Table S1: Characteristic of *S. cerevisiae* isolates and susceptibility data.
